# Retrospective Cohort Analysis of Central Line Associated Blood Stream Infection following Introduction of a Central Line Bundle in a Neonatal Intensive Care Unit

**DOI:** 10.1155/2018/4658181

**Published:** 2018-09-02

**Authors:** Molly Bannatyne, Judith Smith, Malavika Panda, Mohamed E. Abdel-Latif, Tejasvi Chaudhari

**Affiliations:** ^1^Australian National University Medical School, 54 Mills Road Acton, ACT 2601, Canberra, Australia; ^2^The Canberra Hospital Department of Neonatology, Building 11 level 2 Centenary Hospital for Women and Children, ACT 2606 Canberra, Australia

## Abstract

**Background:**

Central Line Associated Bloodstream Infections (CLABSI) constitute a leading cause of morbidity and mortality in neonatal populations. There has been an overwhelming increase in the use of evidence-based care practices, also known as bundles, in the reduction of these infections. In this report, rates of CLABSI and central line utilisation were examined following the introduction of a central line bundle in our Neonatal Intensive Care Unit (NICU) at the Canberra Hospital.

**Methods:**

The research undertaken was a retrospective cohort study in which newborn infants admitted to the Canberra Hospital NICU between January 2011 and December 2016 and had a central line inserted were included in the study. Data regarding central line days, bed days, infection rates, and patient demographics were collected before and after the introduction of an intervention bundle. CLABSI rates were calculated per 1,000 central line days for before (2011-2013) and after (2014-2016) the introduction of the bundle. The postintervention period was retrospectively analysed for compliance, with data regarding the completion of maintenance forms and insertion forms collected.

**Results:**

Overall, the results showed a significant decrease in CLABSI rates from 8.8 per 1,000 central line days to 4.9 per 1,000 central line days in the intervention period (p<0.001). Central line utilisation ratio (CLUR: ratio of central line days to bed days) was also reduced between pre- and postintervention periods, from 0.177 (4414/25013) to 0.13 (3633/27384; p<0.001). Compliance to insertion forms and maintenance forms was observed to increase within the intervention period.

**Conclusion:**

The implementation of a central line bundle was effective in reducing both CLABSI rates and dwell time (CLUR) for central venous catheters.

## 1. Introduction

Obtaining secure and reliable vascular access is an integral component of provision of care to patients in the Neonatal Intensive Care Unit (NICU). Central lines are most commonly used to ensure venous access in both sick and premature babies. These lines provide a means through which fluid and medications can be administered, as well as allowing regular blood pressure monitoring and blood sampling, each of which is vital to the growth and survival of patients in the NICU [1, 2]. Central Venous Catheters (CVC) commonly used in the NICU include Peripherally Inserted Central Venous Catheters (PICC), Umbilical Venous Catheters (UVC), and Umbilical Arterial Catheters (UAC). Each of these lines, while essential to provision of a consistent standard of care, poses a risk of Central Line Associated Blood Stream Infections (CLABSI), particularly in extremely premature and low birth weight infants [1].

According to the US Centers for Disease Control and Prevention, a CLABSI are defined as “a primary blood stream infection in a patient that had a central line within the 48-hour period before the development of the blood stream infection, and is not related to an infection at another site” [3]. CLABSI infections usually occur via commensals which migrate directly via the catheter to the blood, with the overriding causative organism being Coagulase Negative* Staphylococcus* (CONS) [4]. These infections contribute to poor patient outcomes and increased individual length of stay, as well as increasing hospital costs [5, 6]. The risk of CLABSI is significantly increased in patients with lower gestational age and birth weight, as they exhibit poor skin integrity, an immature immune system, and require the prolonged use of central lines [6]. As a result, CLABSI are the most common cause of late onset sepsis (LOS) in neonates and thus constitute one of the leading causes of both morbidity and mortality in this age group [7]. The Australian and New Zealand Neonatal Network (ANZNN) reports the incidence of late onset sepsis as 4.5%, with CLABSI constituting 69% of these infections [8]. However, the incidence of CLABSI varies between NICUs, ranging between 1.6 and 15 per 1,000 central line days [9–11].

The significant burden of CLABSI in neonates suggests that prevention is vital to the reduction of morbidity, mortality, and financial burden. The Australian National Safety and Quality Health Service Standards suggest that prevention and control of Healthcare Associated Infection (HAI) is a key component [12]. This idea highlights the importance of seeking prevention for infections such as CLABSI. CLABSI rates have been shown to reduce with the use of healthcare intervention “bundles” [13–15]. These bundles are defined by the Institute for Healthcare Improvement (IHI), as a “small straightforward set of evidence-based practices that, when performed collectively and reliably, have been proven to improve patient outcomes” [16]. Most NICUs have modified and adopted the original five components of the IHI central line bundle; hand hygiene, maximal barrier precautions during insertion, chlorhexidine skin antisepsis, optimal site selection, and maintenance of the central line through daily review.

Investigation of CLABSI at The Canberra Hospital (TCH) in 2012-2013 showed one of the highest rates among each of the NICUs in New South Wales and the Australian Capital Territory [7]. In response, current practices were audited and a new central line bundle policy was introduced in 2014 in attempt to lower this statistic. The aim of this study was to report the impact of the CLABSI prevention bundle on rates of CLABSI in neonates at TCH NICU, thereby determining its effectiveness and areas for further refinement.

## 2. Methods

### 2.1. Project Setting and Design

Use of the central line bundle commenced in TCH NICU in January 2014. Retrospective assessment of its impact occurred via comparing CLABSI rates between two distinct study periods, January 2011 to December 2013 as the baseline preintervention group and January 2014 to December 2016 as the intervention group. This review was approved by the Canberra Hospital Human Research Ethics Committee (ETHLR.18.188).

### 2.2. Quality Improvement Process and Intervention

**Figure 1:**
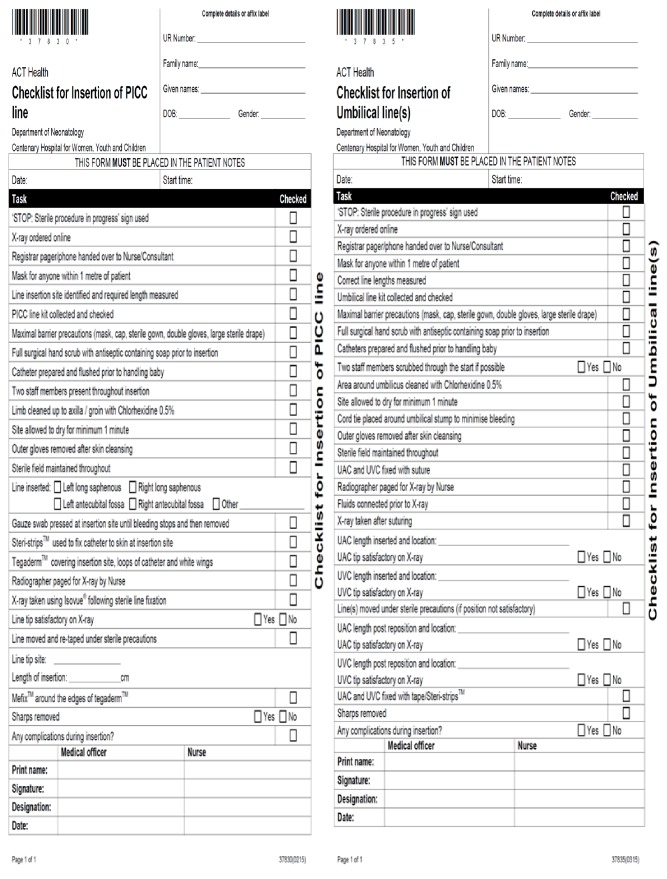
Insertion checklist for peripherally inserted central catheters and umbilical lines.

**Figure 2:**
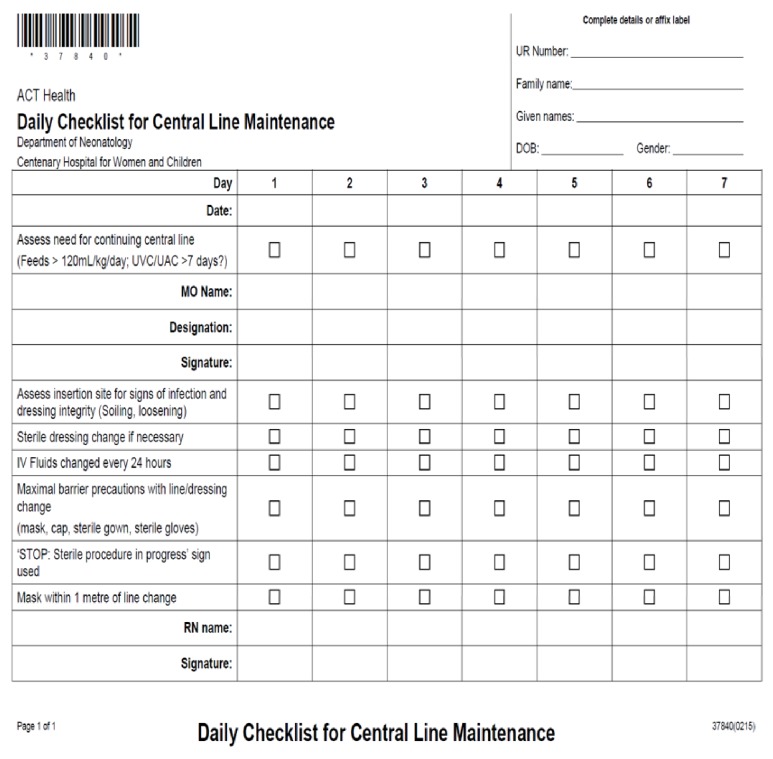
Daily checklist for central line maintenance.

In 2013 the research team commenced both analysis and reporting of CLABSI at regular neonatal mortality and morbidity meetings. Following recognition of a high rate of CLABSI in the NICU, a central line bundle was proposed [7]. This bundle was developed from current evidence and literature reviews [1–8, 10, 11, 13–15]. The final bundle included a number of interventions:

1. Introduction of insertion and maintenance checklists (Figures 1 and 2)
2. Intensive education of medical and nursing staff
3. Implementation of an exclusive central line cart which contained consolidated items required for central line insertion
4. Encouraging nursing staff to enforce the checklist and stop procedure if sterility was breached.
5. Placing a ‘STOP' sign outside of patient rooms during procedures
6. Ensuring that maximal barrier precautions are taken during procedures
7. Ensuring that two people are scrubbed during the procedure
8. Only senior medical officers to insert central lines in babies
9. Introduction of digital radiology plate for final position confirmation
10. Positive reinforcement and celebrating success
11. Regular data collection and presentation

CLABSI prevention is a complex process and contamination can occur at multiple steps. Failure to maintain sterility at any of the steps can cause CLABSI. By combining several evidence-based practices into a single bundle, the probability of implementation and adherence to each of the individual components is increased, resulting in better patient outcomes. As such, this study was not designed to show the relative contributions of the 11 interventions.

### 2.3. Data Collection

Data was extracted for each patient who had a central line placed during their NICU admission from January 2011 until December 2016 using the electronic neonatal database system (NICUS), pathology result system (CIS), and hospital medical records (CRIS). The data obtained included clinical characteristics, CVC use, details of bloodstream infection, central line days, and bed days. The central line days were calculated as the total number of days a central line was in place for each patient in the NICU. This count was performed daily and each patient with a central line, regardless of whether there were single or multiple vascular access points, and was counted as a central line day, with bed days being measured similarly. CLABSI rates were calculated as the number of CLABSI x1000/central line days. CLABSI rates were analysed as part of birth weight cohort groups. These groups included less than 750 g, 751-1000 g, 1001-1500 g, and 1501-2500 g and more than 2500 g. Central line utilisation ratio (CLUR) was calculated as the number of central line days/number of patient days. Infections were coded by the neonatologist using standard ANZNN and NICUS definitions for neonatal infections [17].

Compliance data was extracted from insertion and maintenance checklists completed for each central line inserted during the intervention period. The data obtained included form completion and the number of maintenance days recorded. Maintenance was then reported as a maintenance ratio, as calculated by number of maintenance days/number of central line days. This process was repeated for nurses and doctors as subsets of compliance.

### 2.4. Statistical Analysis

All data was managed using an electronic spreadsheet (Excel 2016; Microsoft). CLABSI rates and CLUR for both pre- and postintervention periods were compared using comparison of rates function on Medcalc software, version 15.8. A p value <0.05 was considered significant.

## 3. Results

### 3.1. Patient Characteristics

Patient demographic characteristics are shown in Table 1. There were no significant differences between the baseline and intervention groups for any of the recorded parameters.

### 3.2. CLABSI

**Figure 3:**
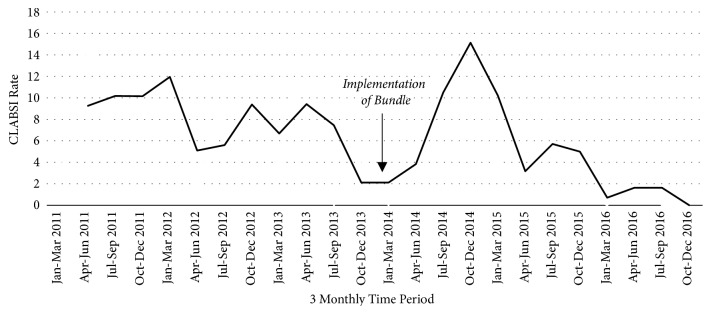
Run chart of Central Line Associated Blood Stream Infections (CLABSI) per 1,000 central line days 2011-2016.

**Figure 4:**
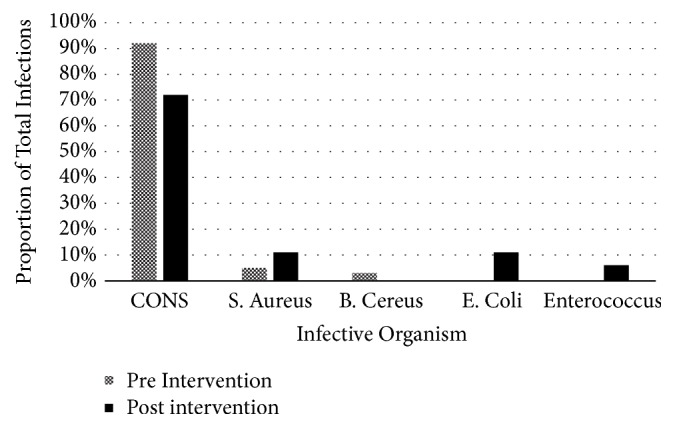
Comparison of contributions of causative organisms in preintervention (2011-2013) and postintervention (2014-2016) groups.

Figure 3 shows the decline in CLABSI between the preintervention period 2011-2013 and the intervention period 2014-2016. There were 18 CLABSI in the intervention period compared to 39 in the preintervention period. Most prominently, CLABSI rates have been following a negative trend since the implementation of central line bundles. Figure 3 also details the 3 monthly CLABSI rates in both the preintervention and the intervention periods. Since July 2014, this shows an overarching negative trend. There was an initial peak in 2014 after implementation of the bundle. Review of individual infection cases within this period revealed a notable number of patients suffering from necrotising enterocolitis, requiring longer CVC dwell times.  Figure 4 portrays the contribution of differing organisms to CLABSI in both research groups. Of the CLABSI in the preintervention group, 36 (92%) were positively cultured for CONS, 2 (5%) for Staphylococcus aureus, and 1 (3%) for Bacillus cereus. Of the 18 CLABSI in the intervention group, CONS was positively cultured in 13 (72%) cases, Staphylococcus aureus for 2 (11%), Escherichia coli for 2 (11%), and 1 (6%) for Enterococcus.

Overall, CLABSI rates saw a significant decrease between preintervention and intervention groups. Rates dropped from 8.8 per 1000 central line days to 4.9 per 1000 central line days (p<0.001). This trend is also replicated for each of the birth weight groups, as shown in Table 2, except for the 751-1000 g weight group which saw a significant increase in CLABSI infection.

### 3.3. Central Line Utilisation Ratio

The central line utilisation ratio (CLUR) was used as a measure of how long the central lines dwelled in situ. Results showed a significant decline in CLUR between the baseline and intervention periods (p<0.001).

### 3.4. Compliance

**Figure 5:**
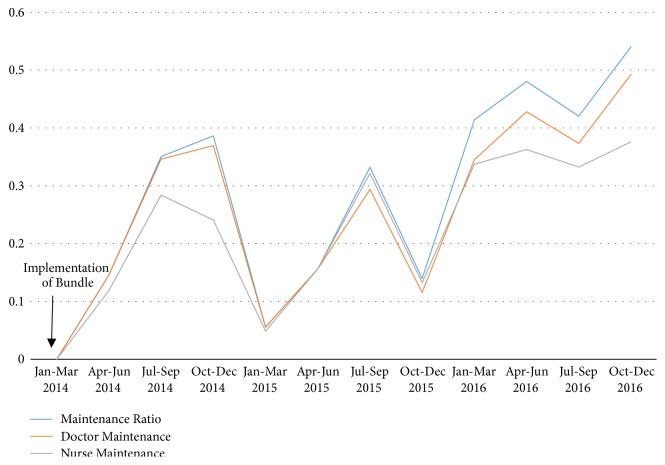
Maintenance ratio during implementation period of a central line bundle. Maintenance represented as a ratio of maintenance days/amount of days in which central lines were in place.

Completion of PICC and UAC/UVC insertion checklists (Figure 1) was used as a measure of compliance to insertion procedures. Within the intervention period, compliance to insertion bundles increased from 29.5% to 80.5% and 97.7% in the following years. The maintenance ratio was used as a measure of how strictly the maintenance checklists (Figure 2) were adhered to in the first years following implementation of the bundle.  Figure 5 shows the trends in overall maintenance ratio during the 2014-2016 period, as well as individual maintenance ratios for doctors and nurses. This trend shows an overall increase in maintenance ratio throughout the entire intervention period, for all groups. However, there are intermittent periods of decline in the overall maintenance ratio throughout the time period. These intermittent declines are replicated for doctors and nurses.

## 4. Discussion and Conclusion

In extremely premature and vulnerable populations, HAIs remain an important issue worldwide. This report aimed to investigate the effects of implementing an interventional bundle on the rates of these infections in a particularly at risk population in TCH NICU. While the impact of introducing a care bundle has been demonstrated in this report for a single NICU, the results suggest that there is potential for similar substantial impact worldwide. By generalising the care bundle to other facilities with similar resources, the impact on infection rates could be observed on a much larger scale [14, 18, 19].

The major findings of this study suggested that implementing a multifaceted intervention bundle could significantly change clinical practice surrounding the use of CVCs. This change in protocol translated into significant decreases in clinical complications associated with CVC use, namely, the reduction in CLABSI rates. The results showed that the reduction in CLABSI was significant for all birthweight categories, with the exception of the 751-1000 g weight group. This suggests that while the risk of contracting CLABSI is known higher in patients of both younger gestational age and lighter birthweight, the effects of implementing a care bundle are relevant for all patients in the NICU. The significant increase in CLABSI rates within the 751-1000 g birthweight category is inconsistent with similar studies conducted on a larger scale [14, 18, 19].

Despite the overall significant reduction of CLABSI rates, there was an initial peak in 2014 after implementation of the bundle. This may be the result of increased necrotising enterocolitis in our unit during that period necessitating increased central line insertions, as well as dwell time due to short gut. As such, their risk of contracting CLABSI was greater than the baseline NICU population. Additionally, the initial implementation period for the central line bundles was accompanied by a poor compliance rate. The combination of these factors contributed to an increase in infection rates in early 2014. However, this prompted a focus on education and auditing of infections at subsequent multidisciplinary meetings. As a result, the NICU experienced both an increase in compliance to insertion protocols and a significant reduction in CLABSI rates in the following years. Thus, it can be inferred that the implementation of the bundle in combination with effective education and review is successful as a preventative measure against CLABSI [14, 18, 19].

The majority of infection for both preintervention and intervention groups was attributable to positive blood cultures for CONS. These organisms are widely recognised to be associated with CLABSI due to their tropic preference as a skin commensal [4]. Insertion of CVCs provides a thoroughfare through which these bacteria can migrate to the bloodstream to cause widely disseminated bloodstream infection. The results suggest that the implementation of a central line bundle led to a decrease in overall contribution of CONS to CLABSI. Thus, it can be inferred that the bundles were capable of reducing the direct exposure of the bloodstream to skin commensals such as CONS, thereby reducing the overall burden of CLABSI.

Overall compliance to maintenance forms (Figure 2) has risen since the implementation in 2014. Despite this, there were periods of fluctuation in adherence, for both doctors and nurses. The most significant increase in maintenance ratios occurred within the first six months of implementation. This is most likely attributable to the initial implementation of the bundle being accompanied by education on the importance of the intervention in improving infection rates. Additionally, examination of the insertion forms completed within this period showed an overall increase in completion rate for both UAC/UVC and PICC lines. With increased compliance to these insertion protocols, the NICU has seen significant reduction in CLABSI rates. This suggests that while compliance to insertion procedures has seen a notable increase, there remain areas for improvement in complying to maintenance procedures that were introduced. Thus, with a particular focus on increasing adherence to maintenance criteria, the NICU could see further improvement in both the infection rates and the efficiency and uniformity with which procedures are carried out.

The CLABSI prevention bundle now forms part of our normal clinical practice. Infection data is collected and coded via neonatologists and audit officers, with CLABSI rates being presented to the entire unit at 3 monthly M and M meetings. Comparative infection rates among different NICU's are also presented at these meetings. A root cause analysis (RCA) is performed following any new CLABSI infection to identify potential breach in bundle delivery or any preventable issues. Compliance to insertion and maintenance bundles is regularly audited and fed back. With regular data collection and review, lapses in the remainder of the bundle could be identified and actioned as required, thus contributing to a sustained reduction in overall CLABSI rates.

Limitations of the study include a small dataset, particularly due to the study population being restricted to a single NICU facility. This could provide a possible explanation for the discrepancy observed in our 751-1000 g dataset compared to studies conducted on a larger scale [14, 15, 18, 19]. However, the overall observed reduction in infection rates is consistent with similar outcomes for prevention bundles examined on a much larger scale [14, 15, 18, 19]. Thus, the results of our study suggest that a significant reduction in CLABSI could be pivotal to reducing the burden of HAI in NICUs as well as paediatric and adult ICUs [19]. Due to the intrinsic nature of a retrospective cohort study, this analysis is prone to bias. However, examination of demographics in both preintervention and intervention groups showed no significant differences, thus minimising this bias. The reported maintenance rates in the above data suggest poor compliance to maintaining the implemented bundle. As such, it is difficult to determine whether there are other confounding factors influencing the overall reduction in infection rates. Despite this reported compliance to maintenance procedures, the NICU still experienced a significant reduction in CLABSI rates. This suggests that the reduction observed could be replicated on a much larger scale with increased adherence to protocol. In order to confirm that the reduction in CLABSI can be attributed entirely to the implementation of quality improvement and bundle processes, ongoing audit will be necessary.

Although the global burden of CLABSI among NICUs is declining, CLABSI continus to pose a significant risk on an individual patient level, while continuing to lengthen hospital stay and associated financial burden [5, 6, 20, 21]. As such, the reduction of HAI such as these remains a major aim of hospitals worldwide [12]. This study demonstrated that bundles of intervention can have substantial impact within single facility, suggesting that generalisation of bundles to other institutions could produce a more significant and widespread effect. It thus follows that similar procedures could be adopted in paediatric and adult intensive care units to achieve comparable results. In future practice, maintenance of achievements will be paramount to achieving overall reduction in HAI. This will be achieved via ongoing education and review, as well as innovation of new interventions to further reduce infection.

## Figures and Tables

**Table 1:** Characteristics of Infants with central lines Inserted in baseline (2011-2013) and intervention (2014-2016) groups.

**Characteristic**	**Baseline (n=406)**	**Intervention (n=331)**
Male sex, %	55.4	57.4
Gestation, median (IQR), weeks	33(29-38)	34(28-39)
Birthweight, median (IQR), g	2970(1695-4245)	3100(1800-4400)
Antenatal Steroids (n)	172	139

**Table 2:** Central Line Associated Bloodstream Infections (CLABSI) per 1,000 central line days for different weight categories.

**Birthweight Category (g)**	**Baseline Group 2011-2013**	**Intervention Group 2014-16**	**P-Value**
<750	16.8	12.0	<0.001^*∗*^
751-1000	8.3	9.0	<0.001^*∗*+^
1001-1500	8.5	3.9	<0.001^*∗*^
1501-2500	6.4	1.8	<0.001^*∗*^
>2500	6.7	3.1	<0.001^*∗*^

A P value <0.05 was considered significant. All categories show a statistically significant decrease unless otherwise specified.

+ Statistically significant increase.

## Data Availability

The data used to support the findings of this study are available from the corresponding author upon request.

## Appendix

See Figures 1, 2, 3, 4, and 5 and Tables 1 and 2.

## References

[1] Janes M., Kalyn A., Pinelli J., Paes B. (2000). A randomized trial comparing peripherally inserted central venous catheters and peripheral intravenous catheters in infants with very low birth weight. *Journal of Pediatric Surgery*.

[2] Ainsworth S. B., Clerihew L., McGuire W. (2007). Percutaneous central venous catheters versus peripheral cannulae for delivery of parenteral nutrition in neonates. *Cochrane Database of Systematic Reviews*.

[3] O'Grady N. P., Alexander M., Burns L. A. (2011). Guidelines for the prevention of intravascular catheter-related infections. *American Journal of Infection Control*.

[4] Salzman M. B., Isenberg H. D., Shapiro J. F., Lipsitz P. J., Rubin L. G. (1993). A prospective study of the catheter hub as the portal of entry for microorganisms causing catheter-related sepsis in neonates. *The Journal of Infectious Diseases*.

[5] Dimick J. B., Pelz R. K., Consunji R., Swoboda S. M., Hendrix C. W., Lipsett P. A. (2001). Increased resource use associated with catheter-related bloodstream infection in the surgical intensive care unit. *JAMA Surgery*.

[6] Warren D. K., Quadir W. W., Hollenbeak C. S., Elward A. M., Cox M. J., Fraser V. J. (2006). Attributable cost of catheter-associated bloodstream infections among intensive care patients in a nonteaching hospital. *Critical Care Medicine*.

[7] Bowen J. R., Callander I., Richards R., Lindrea K. B. (2016). Decreasing infection in neonatal intensive care units through quality improvement. *Archives of Disease in Childhood - Fetal and Neonatal Edition*.

[8] Chow S. S. W., Le Marsney R., Hossain S., Haslam R., Lui K. (2016). *Report of the Australian and New Zealand Neonatal Network 2014*.

[9] Aly H., Herson V., Duncan A. (2005). Is bloodstream infection preventable among premature infants? A tale of two cities. *Pediatrics*.

[10] Cartwright D. W. (2004). Central venous lines in neonates: a study of 2186 catheters. *Archives of Disease in Childhood. Fetal and Neonatal Edition*.

[11] Milstone A. M., Reich N. G., Advani S. (2013). Catheter dwell time and clabsis in neonates with piccs: a multicenter cohort study. *Pediatrics*.

[12] Australian Commission on Safety and Quality in Healthcare (ACSQHC) Improvement guide standard 3: preventing and controlling healthcare associated infections. http://www.safetyandquality.gov.au/wp-content/uploads/2012/10/Standard3_Oct_2012_WEB.pdf.

[13] Blot K., Bergs J., Vogelaers D., Blot S., Vandijck D. (2014). Prevention of central line-associated bloodstream infections through quality improvement interventions: a systematic review and meta-analysis. *Clinical Infectious Diseases*.

[14] Schulman J., Stricof R., Stevens T. P. (2011). Statewide NICU central-line-associated bloodstream infection rates decline after bundles and checklists. *Pediatrics*.

[15] McMullan R., Gordon A. (2016). Impact of a central line infection prevention bundle in newborn infants. *Infection Control & Hospital Epidemiology*.

[16] Institute for Healthcare Improvement (IHI) How-to guide: Prevent central line-associated bloodstream infection. http://www.ihi.org/resorces/Pages/Tools/HowtoGuidePreventCentralLineAssociatedBloodstreamInfection.

[17] Australian and New Zealand Neonatal Network ANZNN 2018 Data Dictionary. https://anznn.net/Portals/0/DataDictionaries/ANZNN_2018_Data_Dictionary.pdf.

[18] Bizzarro M. J., Sabo B., Noonan M., Bonfiglio M.-P., Northrup V., Diefenbach K. (2010). A quality improvement initiative to reduce central line-Associated bloodstream infections in a neonatal intensive care unit. *Infection Control and Hospital Epidemiology*.

[19] Ista E., Van Der Hoven B., Kornelisse R. F. (2016). Effectiveness of insertion and maintenance bundles to prevent central-line-associated bloodstream infections in critically ill patients of all ages: a systematic review and meta-analysis. *The Lancet Infectious Diseases*.

[20] Dudeck M. A., Weiner L. M., Allen-Bridson K. (2012). National Healthcare Safety Network (NHSN) report, data summary for 2012, device-associated module. *American Journal of Infection Control*.

[21] Jaggi N., Rodrigues C., Rosenthal V. D. (2013). Impact of an international nosocomial infection control consortium multidimensional approach on central line-associated bloodstream infection rates in adult intensive care units in eight cities in India. *International Journal of Infectious Diseases*.

